# Development and evaluation of a German Paediatric Trigger Tool (PAED-TT-GER) for detecting adverse events in hospitalized children

**DOI:** 10.1007/s00431-026-07307-5

**Published:** 2026-08-17

**Authors:** Anthea Peters, Isabel Schulz, Nicole Müller, Matthias Weigl

**Affiliations:** 1 Department of Paediatric Cardiology, University Hospital, Bonn University, Bonn, Germany; 2 Institute for Patient Safety, University Hospital, Bonn University, Bonn, Germany

**Keywords:** Paediatric care, Hospital, Patient safety, Harm, Healthcare quality

## Abstract

Trigger tools (TTs) are highly recommended for structured detection of adverse events (AEs) through retrospective medical record review. However, available general tools require thorough adaptation to paediatric care and local documentation practices to ensure feasibility and reliable AE identification. The aim of this study was to develop a German Paediatric Trigger Tool (PAED-TT-GER) and evaluate its feasibility, applicability, and interrater reliability in a paediatric cardiology inpatient setting, and to describe the characteristics of AEs detected. We conducted a single-centre, retrospective methodological study on a paediatric cardiology general ward at a tertiary university hospital in Germany. PAED-TT-GER was developed in a stepwise process: (1) literature-based trigger extraction, (2) interdisciplinary expert consensus and contextual adaptation, (3) refinement of standardized operational definitions, and (4) pilot testing and interrater reliability assessment. In the main application, 200 electronic inpatient records (inclusion criteria: age < 18 years; length of stay ≥ 24 h; discharge ≥ 30 days before review) were screened. Records with identified triggers underwent secondary physician review to confirm AEs. AE severity was classified using NCC MERP (E–I), and preventability was rated using an established, six-point scale. From an initial pool of 145 candidate triggers, 57 triggers were selected (54 for general wards plus 3 supplementary triggers for potential PICU use). Interrater reliability testing in 27 records showed good agreement (ICC 0.794; 95% CI 0.557–0.906). In the main study cohort with 200 patients, 472 triggers were identified in sum (mean 2.36 per record; range 0–13). A total of 45 AEs were confirmed in 42/200 records (21.0%). Most AEs caused temporary harm requiring intervention (NCC MERP E, 24/45) or prolonged hospitalization (F, 19/45); two events resulted in permanent harm (G, 2/45). Seven AEs (15.6%) were rated as preventable after chart review.

*Conclusion*: PAED-TT-GER is a feasible and reliable instrument for detecting AEs in hospitalized children within a German paediatric context. It enables systematic harm measurement and provides a foundation for paediatric patient safety surveillance and future validation across additional paediatric subspecialties and care settings.

**Table:** 

**What Is Known:** *• Trigger tools enable structured retrospective detection of adverse events in paediatric inpatient care, but their applicability depends on adaptation to paediatric populations and specific healthcare and documentation contexts.* **What Is New:** *• The newly developed German Paediatric Trigger Tool (PAED-TT-GER), comprising 54 general-ward triggers and three supplementary PICU triggers, demonstrated good interrater reliability in a paediatric cardiology setting.*

**Supplementary Information:**

The online version contains supplementary material available at https://doi.org/10.1007/s00431-026-07307-5.

## Introduction

Adverse events (AEs) represent a major challenge to healthcare systems worldwide. Studies suggest that around 12% of all hospital admissions are associated with at least one adverse event (AE), accounting for a substantial proportion of patient harm [1, 2].

Systematic identification of patient harm and monitoring of AEs is fundamental for any improvement efforts for patient safety and healthcare quality. Assessments that draw upon trigger tool–based methods have shown greatest promise in effectively detecting patient harm in clinical practice. Trigger tools (TT) use structured retrospective medical record review and scan for predefined “triggers”—signals that indicate the possible occurrence of an AE, which are subsequently evaluated by trained reviewers to determine whether an AE has occurred [3, 4]. Thus, TT approaches facilitate identification of recurring patterns of harm and underlying systemic weaknesses in clinical processes [5]. Increasing evidence suggests that TT approaches are superior in identification of AEs and detecting patient harm that would otherwise remain unrecognized, particularly in comparison to reporting systems. Previous studies indicate that only approximately 3–7% of events detected using a TT are also reported through routine and voluntary incident reporting systems [3, 5, 6]. Moreover, efficacy, reliability, and applicability of TT approaches increase if pre-defined triggers are tailored to specific clinical specialties and realities of care contexts, as structural and resource-related barriers impede routine clinical application [7].

Given the many inherent complexities and unique hazards involved in paediatric care, TT approaches with adapted triggers that reflect its specific risks and harm patterns are required. Previous studies showed that generic TT approaches (e.g., the Global Trigger Tool, GTT), have limited sensitivity and low positive predictive value in paediatric patients if applied without modification [8–13]. This has also been attributed to the fact that generic triggers inadequately capture paediatric-specific AEs, including neonatal complications, age-dependent infections, and weight-adjusted medication errors [14].

To address these limitations, several paediatric-adapted trigger tools have been developed and validated in recent years, including the Paediatric Trigger Tool (PTT), the Global Assessment of Pediatric Patient Safety (GAPPS), and national adaptations such as the Canadian Paediatric Trigger Tool (CPTT) [12, 13, 15]. These instruments incorporate paediatric-specific triggers and demonstrate substantially improved sensitivity for detecting AEs in children, with reported sensitivities of up to 88% [12, 13]. At the same time, they achieve higher positive predictive values, typically ranging from 16 to 25%, thereby reducing false-positive findings and enhancing clinical relevance [9–11]. Moreover, TT approaches tailored to paediatrics allow for targeted quality improvement initiatives [12, 16].

Although several paediatric TTs have been developed internationally, no TT specifically adapted and validated for use in German-speaking paediatric healthcare settings is currently available. Existing tools cannot simply be translated, as clinical documentation practices, medication use, and healthcare structures differ substantially between countries and healthcare systems. Consequently, a systematic cultural and clinical adaptation process is required to ensure applicability and validity within the target setting. This need is further reinforced by the heterogeneity of paediatric inpatient care, which encompasses multiple subspecialties with distinct risk profiles and AE patterns. For example, paediatric cardiology patients frequently exhibit disease-specific physiological parameters, such as lower target oxygen saturation ranges, which may affect the performance and interpretation of generic trigger criteria. To address these challenges, we conducted a study to develop and evaluate a novel German language paediatric trigger tool (PAED-TT-GER) intended for use across paediatric inpatient care in German-speaking healthcare settings. As a first step in its evaluation, the tool was applied in a paediatric cardiology inpatient population, representing a complex clinical environment with a high potential for AEs. Ultimately, PAED-TT-GER is intended to systematically and reliably assess patient safety in paediatric inpatient care within German-speaking healthcare settings.

Therefore, the objectives of this study were (1) to assess the feasibility, applicability, and interrater reliability of PAED-TT-GER and (2) to describe the characteristics of adverse events identified using the tool.

## Methods

### Study design

A retrospective, single-centre methodological study design was established that included the systematic and step-wise development, adaptation, and testing of a novel paediatric TT for identification of AE in hospitalized children in Germany. Sample sizes were determined based on feasibility considerations and aligned with sample sizes used in comparable paediatric TT development and validation studies.

The study received approval from the Ethics Committee of the Medical Faculty at Bonn University (2024–459-BO). We did not collect personal or identifying information, neither from patients nor from participating clinicians. The study was conducted in accordance with the principles of the Declaration of Helsinki. All data were retrospectively extracted from electronic patient records (local EMR system: ORBIS, Dedalus HealthCare, Bonn, Germany). Chart review was performed in a pseudonymized manner, and all datasets used for analysis were anonymized prior to statistical evaluation.

To ensure comprehensive reporting, we used the STROBE Checklist [17]. Furthermore, to ensure comprehensive and standardized reporting of potential patient harm and AE, we applied the SESAME statement [18].

### Setting and study population

Although PAED-TT-GER was developed for application across paediatric inpatient care, its initial evaluation was conducted on a paediatric cardiology general ward at a tertiary university hospital in Germany. This setting was selected as a first step in the evaluation process, as paediatric cardiology represents a complex clinical environment with a high potential for adverse events. The ward comprises 16 inpatient beds and is staffed by 5 physicians and 27 nurses, providing care for approximately 1100 inpatient admissions annually.

The patient population comprised children admitted for a broad range of indications, including diagnostic and interventional cardiac catheterization procedures as well as medical management of congenital and acquired heart disease. A total of 200 electronic patient records from paediatric inpatients treated between August 2020 and October 2023 were retrospectively reviewed. The records were derived from a previously established dataset and served as a convenience sample for the present study. This dataset had originally been assembled for the evaluation of an educational project on the same paediatric cardiology ward. For that project, 100 admissions were randomly selected from patients participating in the educational programme. Subsequently, 100 additional admissions meeting the same eligibility criteria were selected from the same ward and study period to form a comparison cohort for the original project.

All records fulfilled the predefined eligibility criteria of the original study (age < 18 years, hospital stay ≥ 24 h, and discharge at least 30 days before review). For the methodological evaluation of PAED-TT-GER, all 200 records from the existing dataset were analysed as a single cohort irrespective of their original allocation within the educational project. The unit of analysis was the individual hospital admission. Repeated admissions of the same patient were permitted and analysed as separate admissions, reflecting routine clinical practice in paediatric cardiology, where multiple hospitalisations and interventions are common.

### Eligibility criteria for medical records analysis

To capture comprehensive reporting of patients’ paediatric care, several eligibility criteria for selection of medical record review were established, allowing also identification of potentially delayed AEs such as readmissions or post-discharge complications. Medical records were eligible if they met the following criteria:

- Patient’s age < 18 years
- Admitted to and inpatient treatment on a paediatric cardiology general ward
- Length of stay ≥ 24 h
- Completed hospitalization ≥ 30 days before chart review

No exclusions were made based on completeness of records (i.e. defined as all treatment information accessible after discharge), diagnoses, disease severities, or interventions.

### Step-wise development and application of PAED-TT-GER

We developed PAED-TT-GER based on the conceptual framework of IHI’s GTT. In this framework, an adverse event (AE) is defined as unintended patient harm (i.e. physical injury) by medical care that results in additional monitoring, treatment, prolonged hospitalization, or contributes to death [19]. This definition is consistent with similar TT approaches in paediatric care (e.g. GAPPS); it thus excludes cases and events where unforeseen natural disease progression is observed that cannot be attributed to (missed) care, treatment, or interventions while being admitted [15].

Our development and testing process followed a structured multi-step approach.

#### Step 1: Literature-based trigger identification

A comprehensive review of published paediatric TT and validation studies was performed, based on a recent systematic review of AE in paediatric inpatient care [20]. All triggers reported in international paediatric TT publications were extracted and listed in a table including preliminary title and definitions for further review (available upon request from the corresponding author, exclusively in German language).

#### Step 2: Expert review, consensus, and adaptation

We recruited a multi-professional expert panel consisting of seven physicians (including board-certified specialists in paediatrics and paediatric cardiologists) and six paediatric nurses (who also had ICU experience). We used a convenience sample of local healthcare providers who were approached by the first and second author. We defined subject matter expert (SME) as healthcare providers with deeper knowledge, professional experience, and distinguished practical expertise in paediatric care and safety. If providers nominated themselves after information about study objectives and eligibility, they were informed about the next steps. Due to constrained resources, we aimed for 6–7 SMEs. Prior to start, SMEs were introduced to AE definitions and informed on the intended purpose of the final tool.

SME review used a structured, consensus-based approach. It was conducted in two consecutive focus group sessions, where SMEs collectively reviewed the proposed list of triggers (identified in step 1). Prior to the meetings, all SMEs received this list including preliminary definitions. During the in-person sessions, each trigger definition and description was presented, jointly discussed, reviewed, and, if needed, refined.

SMEs were instructed to review and evaluate each proposed triggers with respect to the following criteria: (1) relevance for paediatric inpatient care, (2) applicability within the German healthcare context, (3) feasibility in routine clinical documentation, and (4) clarity of operational definitions. Consensus among SMEs on trigger inclusion or exclusion was reached through iterative group discussion until agreement was achieved. Triggers deemed redundant, ambiguous, or not applicable to paediatric inpatient care were excluded.

#### Step 3: Finalization of PAED-TT-GER

For all retained trigger items after expert review, respective operational definitions were refined and finalized to ensure consistent application during subsequent chart review (cf., step 4). This final PAED-TT-GER included then a complete trigger list, including definitions and trigger categories.

#### Step 4: Pilot testing in medical record review process and reliability testing

All following PAED-TT-GER applications in medical record review were conducted in accordance with standardized procedures derived from the IHI GTT methodology [19]. First, ten randomly selected records were independently reviewed by two paediatric specialists. This phase served to assess feasibility, refine trigger definitions, and optimize the review process. No modifications to the trigger tool itself resulted from this step; however, it contributed to improvements in the documentation system. Secondly, we assessed interrater reliability across two reviewers with different professional background. Twenty-seven patient records were independently reviewed by one paediatric nurse and one paediatric physician after prior training using standardized training cases. Agreement regarding trigger identification and AE detection was determined.

#### Step 5: PAED-TT-GER application in chart reviews and AE evaluation (main study)

Following pilot testing and reliability assessment, 200 patient medical records were reviewed with the PAED-TT-GER through a trained paediatric physician. Prior training was conducted in accordance with the IHI GTT guidelines and included supervised reviews, pilot reviews, and consensus discussions to resolve uncertainties in the review process. In accordance with the IHI GTT guidelines, review times per patient record were limited to 20 min [19]. In exceptional cases involving particularly complex patient records, reviewers were allowed to exceed this time limit at their discretion. Reviewers were not blinded to information on patient identification. When one or more triggers were identified, the record underwent secondary review by a second paediatric specialist to confirm the presence or absence of an AE and to reach consensus.

Additionally, for each identified AE, we further obtained the following information in course of the records review:

1. *Narrative description of the AE*, a brief description of the event was documented.
2. AE severity was rated using the NCC MERP Index (categories E–I), as applied in IHI’s GTT, whereby category E represented temporary harm requiring an intervention, while category F referred to temporary harm resulting in initial or prolonged hospitalization [19, 21].
3. *Preventability of AE* was assessed using the six-point preventability scale proposed by Wilson et al. [22, 23]. Consistent with this original, we defined preventability as an observed error in management due to failure to follow accepted practice at either an individual or system level, where accepted practice was considered the expected standard of care for the average practitioner or healthcare system managing the condition in question. This definition is consistent with the majority of preventability definitions that emphasize the presence of an identifiable, modifiable cause of harm [24]. Ratings ranged from 1 (virtually no evidence for preventability) to 6 (virtually certain evidence for preventability), with intermediate categories reflecting increasing levels of likelihood of preventability. For further analyses, we collated scale levels 4–6 into the category ‘preventable’.

All ratings on AE severity and AE preventability classifications were assigned after consideration and consensus by both expert reviewers. In case of disagreement, a third expert (patient safety researcher) was involved, until consensus was reached.

### Statistical analyses

Extracted trigger information and event data was inserted into a standardized form in MS Excel (Microsoft, Inc., Redmond, USA). After quality checks of data entries, data was transferred into SPSS Statistics 29.0 (IBM Inc., Chicago, IL, USA). Interrater reliability was assessed using the intraclass correlation coefficient (ICC). Since only two raters (first and second author) consistently rated all records, we deployed a two-way mixed model with average measure estimation (absolute agreement). Descriptive statistics were used to summarize trigger frequencies, AE rates, and distributions of severity and preventability. The significance level was set at *p* < 0.05. All statistical analyses were conducted using SPSS Statistics 29.0.

## Results

### The PAED-TT-GER tool

In the first step, our literature review on potentially available triggers in paediatric care revealed an initial pool of 145 potential trigger items. After expert review, 54 triggers were deemed applicable to paediatric general wards in German-speaking healthcare facilities. In addition, three supplementary triggers were defined to support potential future application in paediatric intensive care settings, resulting in a total of 57 triggers. The final trigger set was structured into six modules: (1) laboratory/microbiology, (2) clinical symptoms, (3) interventional procedures, (4) medication, (5) procedural and care, and (6) ICU module. The full PAED-TT-GER trigger tool is listed in Appendix A1.

### Interrater reliability

Interrater reliability of the PAED-TT-GER was assessed using a randomly selected subsample of 27 patient records that were independently reviewed by two trained reviewers. ICC for count of triggers per case was 0.794 (95% CI 0.557–0.906; *p* < 0.001), indicating good interrater agreement. On average, 60.4% of all reported triggers were identical between both reviewers. Mean count of reported triggers was 1.7 (SD 2.0) for Reviewer 1 and 2.4 (SD 2.9) for Reviewer 2, indicating moderate variability in trigger detection across cases. A Wilcoxon signed-rank test revealed a statistically significant difference (*z* = 2.56, *p* = 0.011), indicating a systematic difference in trigger counts while overall agreement was good.

### Main study: study sample characteristics

In step 5, 200 paediatric cardiology patient records were reviewed. Table 1 reports the cohort’s characteristics. Altogether, this distribution reflects routine inpatient paediatric cardiology care and supports the sample`s representativeness.

**Table 1:** Characteristics of the patient population (*N* = 200)

Characteristics	
Age, in years (Md, IQR)	4 (2–11)
Length of hospital stay, in days (Md, IQR)	2 (2–3)
Sex, *n* (%)	
Female	97 (48.5%)
Male	103 (51.5%)

### Prevalence and types of identified triggers

Across all 200 records, a total of 472 triggers were identified with PAED-TT-GER. Mean number of triggers per patient record was 2.36 (SD 2.32, 95% CI 2.07–2.67, range 0–13). When adjusted for length of stay, this corresponded to a mean rate of 96.4 triggers per 100 patient days (SD 84.7). The distribution of triggers was right-skewed, with the majority of records containing one to three triggers (i.e. 67.4% of reviewed records). In 14.5% of records (*n* = 29), no trigger was reported.

Qualitative analysis of identified triggers revealed a broad spectrum of safety-relevant signals, many of which are commonly encountered in paediatric cardiology patients undergoing cardiac catheterization procedures. Most frequently observed trigger categories included (1) hematomas and vascular access complications (*n* = 100, 21.2%), (2) circulatory (*n* = 64, 13.6%) and respiratory abnormalities (*n* = 48, 10.2%), (3) elevated body temperature (*n* = 35, 7.4%), and (4) abnormal wound or skin findings (*n* = 31, 6.6%). Additionally, frequently observed triggers included abnormal blood gas analyses, unplanned diagnostic procedures, and medication-related signals.

### Adverse events: prevalence, severity, and preventability

In total, 45 AEs were identified after case review; in 40 patient records, one AE was detected, and in one 2 AEs and another one 3 AEs. This equates to 42 paediatric patients who had at least one AE during their care, with a prevalence of 21% of all reviewed admissions (95% CI 15.9–27.2%). This corresponds to a positive predictive value (PPV, i.e. proportion of positive triggers that indicate confirmed patient harm or an AE) of 9.7% (95% CI 6.3–14.6%).

Two physicians then rated the severity of confirmed AEs using the NCC MERP scale. Across the 45 AEs, we obtained the following distribution:

- 24 AEs (53.3%) in category E: temporary harm requiring intervention
- 19 AEs (42.2%) in category F: temporary harm requiring prolonged or additional hospitalization
- 2 AEs (4.4%) in category G: permanent harm

Overall, the majority of AEs were evaluated being temporary, though frequently associated with additional diagnostic or therapeutic interventions. Two cases resulted in permanent harm: one involving a thromboembolic cerebral infarction detected at discharge, and one involving persistent third-degree atrioventricular block following cardiac catheterization. These events represent rare but clinically significant outcomes within the cohort.

The most frequently identified AEs were haematoma or aneurysm formation following cardiac catheterization (*n* = 6), hypotension following interventions (*n* = 5), organizational or care management–related problems (*n* = 5), cardiac arrhythmias following interventions (*n* = 4), oxygen desaturation following interventions (*n* = 3), infections (*n* = 3), peripheral venous catheter–related complications (*n* = 3), and positioning-related pressure injuries (*n* = 3).

Preventability was evaluated for all 45 AEs, with 7 AEs (15.6%) being classified as preventable (i.e. scale levels 4–6 ‘probably to certain’). This suggests that a substantial proportion of AEs were deemed to be potentially preventable (cf., Table 2). Among those seven AEs, three were related to positioning issues, one to early discharge with subsequent readmission, one to a peripheral venous catheter infection, one to a needle stick injury requiring additional blood sampling, and one to anal bleeding likely associated with rectal temperature probe placement.

Finally, we explored potential contingencies between severity and preventability for all AEs (cf., Table 2).

**Table 2:** Severity and preventability evaluations for all detected AEs (*n* = 45)

Preventability					
Severity	No evidence	Low to moderate	Unlikely but not excluded	Probable to certain	*(Total)*
E	5	11	3	5	*24*
F	9	1	7	2	*19*
G	1	1	0	0	*2*
*(Total)*	*15 (33.3%)*	*13 (28.9%)*	*10 (22.2%)*	*7 (15.6%)*	*45*

AD severity was classified according to NCC MERP (E–G). Values are AE counts

Within the highest category of harm (i.e. category G), no patient case was evaluated as preventable. However, preventability was attributed to five category E events and two category F events.

## Discussion

We report the development of the PAED-TT-GER, a novel and customized trigger tool designed to systematically detect AEs in hospitalized children using retrospective medical record review. PAED-TT-GER can be applied in routine clinical documentation and allows identification of a substantial number of safety-relevant signals. Our empirical findings support that PAED-TT-GER is an instrument for measuring patient harm in paediatric inpatient care. Nonetheless, our current evaluation was limited to single paediatric cardiology unit and the observed trigger frequencies likely reflect characteristics of this particular patient population and should, therefore, not be interpreted as representative of general paediatric inpatient care. Thus, future applications should confirm its applicability beyond paediatric cardiology inpatient populations in Germany.

The PAED-TT-GER showed sufficient reliability and performance characteristics in course of this first application. Interrater reliability analyses showed good agreement between both clinical reviewers. Although a statistically significant difference in trigger counts between reviewers was observed, such variability is well described in previous TT applications and reflects the inherent challenges in the course of screening process, potential subjectivity, and dependence on document quality and clarity in the course of retrospective chart review rather than inadequate tool performance [13, 19]. Importantly, the combination of good ICC values and consistencies indicates that reviewers identified similar patterns of harm across records, consistent with previously reported paediatric TT reliability metrics [12, 25]. Nonetheless, future applications and evaluations of PAED-TT-GER should also check for potential differences between reviewers, which may also be due to professional education and training. Although a trained nurse was actively involved in our chart screening process, future investigations should test the tool’s usability and performance when being used by different professionals across the different stages of the review process.

In our sample, we identified a mean of 2.36 triggers per patient record. Moreover, we detected AEs in 21% of reviewed patient admissions. These numbers are comparable to AE rates reported in international paediatric TT investigations, which typically range between 15 and 30% depending on setting and methodology [8, 12, 13, 25]. Given that a large proportion of patients underwent cardiac catheterization, the baseline risk for AEs in this cohort may be inherently elevated, as invasive and surgical procedures represent well-established risk factors for potential patient harm [1, 6, 20]. Although upcoming applications of the tool should further scrutinize its performance, the observed rates suggest that PAED-TT-GER is sufficiently sensitive to capture relevant patient harm in a paediatric cardiology inpatient population with heterogeneous age distribution and disease complexity. Nonetheless, our point estimates of positive predictive value should be interpreted carefully since this outcome and performance metric is dependent upon several parameters of the clinical population and surveyed sample, i.e. prevalence dependent. Future applications should therefore test its performance characteristics across different clinical settings and patient populations.

Concerning its clinical relevance, most AEs identified using the PAED-TT-GER were classified as temporary harm requiring intervention or prolonged hospitalization (i.e. NCC MERP categories E and F). This finding resonates well with prior paediatric studies demonstrating that the majority of inpatient harm in children is mild to moderate in severity but nevertheless clinically relevant due to associated diagnostic, therapeutic, and organizational consequences [8, 20]. Eventually, we identified two AEs that were classified within the permanent harm category. Both events were fully documented in patients’ medical records and were clinically recognized during routine care. Importantly, standardized post-procedural monitoring protocols, including mandatory ECG assessment prior to discharge and routine neurological examination before discharge, were already implemented at the time of occurrence. Therefore, these AE cannot be retrospectively attributed to identifiable process deficiencies. Rather, we assume that both AE represent rare but known hazards within the risk spectrum of paediatric cardiology care. This AE identification through the PAED-TT-GER does not suggest system failures but demonstrates that even within established safety structures, residual patient risks remain. This distinction between harm and preventability is essential when interpreting TT findings in highly specialized clinical settings.

Therefore, we also evaluated the preventability of the AE. This assessment warrants careful interpretation since all information is obtained retrospectively. Specifically, we relied solely on information available in routine clinical documentation which, ultimately, can be subject to uncertainty and potential misclassification. Nevertheless, a substantial proportion of detected AEs were deemed to be potentially preventable, with 15.6% of AEs being classified as certainly or probably preventable and another 22.2% as possibly preventable, adding up to 38% of the AEs. Although this proportion was observed in a highly selected paediatric cardiology cohort and should therefore not be generalized to other inpatient populations, it is consistent with previous reports indicating that a considerable proportion of paediatric inpatient AEs may be preventable. Our findings are in line with similar investigations reporting preventability estimates ranging from approximately 30 to over 60% in paediatric inpatient settings [12, 13, 20]. To this end, our findings underscore again the importance of systematic harm detection as a prerequisite for targeted quality improvement initiatives.

Lastly, we also investigated the actual nature of repeatedly observed AEs, with vascular access complications, circulatory and respiratory events, and post-interventional complications being identified most frequently. Although we used a customized trigger list within a selected paediatric inpatient sample, this breadth of potentially reoccurring AEs reflects the multidimensional nature of paediatric patient harm and is comparable to the similar investigations on AEs described in other paediatric TT investigations [13, 25].

### Contributions of PAED-TT-GER

Concerning potential contributions to the evidence base, a key strength of this novel PAED-TT-GER lies in its systematic adaptation to paediatric inpatient settings within the German healthcare system. Our step-wise, consensus-based development process involving an interprofessional expert panel of paediatric specialists with profound experience ensured that selected triggers were clinically relevant, clearly defined, and feasible within local documentation practices. This approach mirrors best practices described in international TT development studies and supports both methodological rigor and contextual applicability [12, 13]. Furthermore, the use of standardized operational definitions and a structured review process contributed to good interrater reliability to safeguard reliable assessment, particularly, where clinical documentation and harm patterns may differ substantially from adult care [19, 25].

Lastly, to the best of our knowledge, no TT-based data on AE and patient harm have been reported specifically from paediatric cardiology inpatient settings. While several trigger tools have been developed for general paediatric inpatient care as well as specialised settings such as neonatal and paediatric intensive care units, evidence regarding their application in paediatric cardiology remains limited. The present study therefore provides initial data on the use of PAED-TT-GER in this complex paediatric subspecialty, while the tool itself was developed for broader application across paediatric inpatient care.

### Limitations

Several limitations should be acknowledged. First, this study was conducted at an academic university hospital in Germany, which may limit generalizability to other paediatric settings. In addition, the study population was derived from an existing dataset that had originally been assembled for the evaluation of an educational project and therefore represented a convenience sample rather than a random sample of all eligible admissions. Consequently, some degree of selection bias cannot be excluded, and the study population may not fully reflect the overall paediatric cardiology inpatient population. Furthermore, the initial evaluation of PAED-TT-GER was performed exclusively in a paediatric cardiology population. Although this setting represents a complex patient population with a substantial risk of adverse events, the performance of the tool may differ in other paediatric subspecialties and general paediatric wards. Future studies should therefore evaluate PAED-TT-GER across a broader range of paediatric inpatient settings and patient populations. However, particular attention was paid during the tool development process to ensure that trigger definitions were broadly applicable and usable also across other paediatric inpatient settings, i.e. and thus not restricted to paediatric cardiology. Finally, although our study population reflects the heterogeneity of a paediatric cardiology ward, further validation in other paediatric subspecialties and care settings is required.

Second, as with all retrospective chart review methods, the performance of the PAED-TT-GER depends on the completeness and quality of clinical documentation; e.g. AEs may not have been detected due to poor or incomprehensible documentation [8, 19]. The manual review process is subject to interrater variability. In addition, the high resource requirements of TT reviews through experienced specialists further limit its feasibility in paediatric care, particularly in healthcare systems with constrained staffing and time resources [14]. Retrospective chart review requires specifically trained personnel—typically nursing staff for trigger identification and physicians for assessing severity and causality of adverse events—posing a considerable organizational and personnel burden in routine clinical practice [7].

Third, the screening performance of this novel tool should be further investigated. Our study did not assess the tool’s sensitivity or specificity against an external criterion. However, the absence of a universally accepted reference standard for AE detection is a well-recognized limitation of patient safety research and applies to all trigger-based methodologies [19, 20]. Consequently, several authors recommend combining trigger-based record review with additional data sources to optimize AE detection and strengthen patient safety monitoring [4, 26]. For example, concurrent data from voluntary incident reports might broaden the picture on underlying risks and harm. Notably, during our study period, only a single incident report (via the hospital-internal CIRS platform) was submitted from the paediatric cardiology ward, despite the substantially higher number of AEs identified through PAED-TT-GER. This observation further illustrates the complementary nature of trigger tool methodology and voluntary reporting systems in patient safety surveillance. Moreover, we acknowledge that we did not test for interrater agreement over time as well as on AE preventability and severity evaluations. Future applications should also test the impact of training and experience as well as professional background on tool’s performance characteristics [27]. Altogether, our findings should be conceived as first insights into the tools’ usefulness, yet future applications should strive to further test PAED-TT-GER’s performance and extend its validity.

### Implications for practice and future research

This novel PAED-TT-GER offers a feasible and reliable method for systematic detection of AE in paediatric inpatient care and may serve as a valuable tool for patient safety surveillance, education, and quality improvement initiatives. TT have been shown to identify substantially more AE than voluntary incident reporting systems and therefore provide a more comprehensive picture of patient harm [19, 25].

Concerning future research, we suggest that the PAED-TT-GER should be evaluated in other paediatric settings, beyond paediatric cardiology patients and with particular focus on general paediatric wards and intensive care units. A viable next step could be its application within a prospective trial across different paediatric care settings. Further avenues for future investigations include its usefulness for integration into routine quality management processes as well as for implementation as electronic TT in automatic scanning of electronic patient records. Moreover, the tool provides a methodological foundation for hypothesis-driven studies examining safety endpoints of specific care models or safety interventions.

## Conclusion

In summary, the PAED-TT-GER is a feasible, reliable, and clinically meaningful paediatric trigger tool for detecting AE in hospitalized children. By combining established TT methodology with context-specific adaptation, it contributes an important instrument for advancing patient safety measurement in paediatric care and provides a solid foundation for future patient safety research.

## Supplementary Information

Below is the link to the electronic supplementary material.

ESM 1 (DOCX 23.2 KB)

## Data Availability

The complete PAED-TT-GER trigger tool is provided in Appendix A1 of this article. The comprehensive list of all triggers extracted from international paediatric trigger tool publications, including preliminary titles and definitions used during the development process, is available from the corresponding author upon reasonable request (German language version only). The datasets generated and analysed during the current study are not publicly available due to patient privacy regulations and ethical restrictions but are available from the corresponding author upon reasonable request and subject to approval by the responsible ethics committee.

## Abbreviations

AE: Adverse event
CI: Confidence interval
CPTT: Canadian Paediatric Trigger Tool
EMR: Electronic medical records
GTT: Global Trigger Tool
GAPPS: Global Assessment of Pediatric Patient Safety
IHI: Institute for Healthcare Improvement
IQR: Interquartile range
Md: Median
NCC MERP: National Coordinating Council Medication Error Reporting and Prevention
PAED-TT-GER: German Paediatric Trigger Tool
PICU: Paediatric intensive care unit
PTT: Paediatric Trigger Tool
SD: Standard deviation
SPSS: Statistical Package for the Social Sciences
TT: Trigger tool
