# Supplementary material for: Development and evaluation of a German Paediatric Trigger Tool (PAED-TT-GER) for detecting adverse events in hospitalized children
Source: Eur J Pediatr. 2026 Aug 17;185(9):672. doi: 10.1007/s00431-026-07307-5 (PMC13481396; doi:10.1007/s00431-026-07307-5)
Supplement: Supplementary file 1 [file 431_2026_7307_MOESM1_ESM.docx]

Appendix XX:

Table XX: Trigger Tool a German Paediatric Trigger Tool (PAED-TT-GER) for Detecting Adverse Events in Hospitalized Children

| **Module** | **Trigger – Definition** | **Trigger – Description (short)** |
| --- | --- | --- |
| **Laboratory/ Microbiology** | Abnormalities in blood count (including anaemia (drop in Hb), thrombocytopenia (DIC), leucocytosis, leukopenia, thrombocytopenia, neutropenia) | Haemoglobin decrease > 2 g/dL from baseline (consider relative change in cyanotic congenital heart disease), drop in haematocrit. Anaemia due to bleeding, postoperative status, volume overload, iron deficiency, as well as indication of infection, sepsis, immunological/haematological condition, platelet consumption, medications |
|  | Pathological blood gas analysis (acidosis, alkalosis, drop in Hb, increase in lactate, electrolyte imbalances, blood glucose abnormalities) | Indication of infection, volume depletion, anaemia, hypoglycaemia, pH shift due to vomiting, diarrhoea, medication-related causes, anaerobic metabolic state, electrolyte intoxication |
|  | Increase in infection markers (CRP, IL-6, PCT) | Non-physiological increase in infection markers: indication of infection, not postoperative |
|  | Increase in transaminases, bilirubin (including required treatment) | Medication-related, parenteral nutrition, organ damage, infection |
|  | Rising renal retention parameters (e.g., creatinine, urea) | Medication-related, volume depletion, renal damage, multi-organ failure |
|  | Coagulation abnormalities: PTT, anti-Xa, INR, ACT | Medication error, bleeding, risk of thrombosis, ECMO |
|  | Unexpected increase in D-dimers | Thrombogenic, inadequate anticoagulation (Caution: not postoperative) |
|  | Unexpected increase in CK/CK-MB, troponin | Injury to the coronary vessels, rhabdomyolysis, trauma-related, cardiac (Caution: not postoperative) |
|  | Positive microbiological finding | Indication of (nosocomial) infection – not detected at admission. Wound infection, pneumonia including VAP, UTI with indwelling urinary catheter, MRSA, VRE, Clostridia. |
|  | Positive virological finding | Indication of (nosocomial) infection – not detected at admission. Wound infection, pneumonia including VAP, UTI with indwelling urinary catheter… |
|  | Hb above target value | Dehydration, hyper transfusion (> 16.5) |
| **Clinical Symptoms** | Elevated body temperature, clinical indication of infection | Vomiting, diarrhoea, dysuria, cough, runny nose, hoarseness, redness/overheating, positive auscultation finding: indication of infection (not otherwise specified, >3 days after admission), inadequately treated infection, insufficient hygiene measures, prolonged indwelling foreign body/device (Caution: no perioperative/intensive care warming) |
|  | Extravasation, necrosis, hematoma, thrombophlebitis, local tissue findings | Tissue damage due to inserted foreign material (peripheral IV, CVC, cardiac catheter (thrombophlebitis, DVT, embolism, vessel occlusion)), positioning-related, oedema |
|  | Pressure injury | Intraoperative, postoperative, inadequate nursing care (positioning-related) |
|  | Urinary retention/bladder distension | Medication-related, postoperative, organic in origin, mechanical in origin, infectious |
|  | Neurological abnormalities/abnormal imaging findings | Paresis, anisocoria, lateral asymmetry, newly onset seizures > indication of stroke, bleeding, intracranial pathology, blood glucose imbalance |
|  | Rash, itching | Allergy-related due to food, medications, cholestasis, anaphylaxis |
|  | Abnormal skin or wound findings | Wound dehiscence, wound infection, secondary bleeding, fistula formation, blistering, burn (most likely intraoperative) |
|  | Circulatory compromise/failure | Hypotension, arrhythmia, bradycardia, tachycardia > medication-related, volume depletion, allergy-related, overdose, analgesia-related, electrolyte disturbance, temperature-dependent |
|  | Respiratory compromise/failure | Insufficient respiratory drive, stridor, prolonged mechanical ventilation, positive auscultation finding, hypoxia (10% below individual baseline) > indication of residual anaesthesia, medication-related cause, inadequate weaning, delirium/withdrawal, effusion, aspiration, pneumothorax, oedema, infiltrate |
|  | Withdrawal/delirium (confusion, reduced compliance, hallucinations, scoring) | Medication-related, inadequate orientation due to lack of visual aid, missing hearing aid, unfamiliar environment |
|  | NEC | Evidence of intestinal gas in the bowel wall, free air in the abdomen, bubbles in the portal vein/liver, therapeutic meropenem/vancomycin > insufficient intestinal perfusion |
|  | Obstipation | Including required medical treatment > medication-related, volume depletion, inadequate prophylaxis |
|  | Unanticipated death |  |
|  | Elevated pain level | Increased (additional) doses of analgesics required, indication of inadequate analgesia, complications, delirium/withdrawal |
|  | Renal impairment/failure | Dialysis, anaemia, oligo-/anuria, pathological ultrasound finding (urinary obstruction), infection, elevated retention parameters |
|  | Haemorrhage, bleeding | Coagulation abnormalities, perioperative, traumatic, ulcer-related |
| **Interventional procedures** | Injury to organ structures in the context of invasive procedures (CVC insertion, drain placement, peripheral IV insertion…), complications of indwelling lines/devices | Bleeding, pneumothorax, extravasation, organ perforation |
|  | Unplanned intervention (drain placement, CVC insertion, endotracheal tube repositioning) | Effusion, retention, malposition |
|  | Blood transfusion/administration of blood products | Active bleeding, drop in Hb, volume depletion, hypoxia, resuscitation, shock, postoperative, chronic anaemia |
|  | CPR, emergency call | Cardiorespiratory failure |
|  | Unplanned radiological imaging (X-ray, ultrasound, CT, MRI) | Effusion, oedema, retention, air inclusions, pneumothorax, bleeding, thrombosis, stenosis |
| **Medication** | Antibiotic therapy | Indication of infection (nosocomial), indication of wound infection, perioperative, abnormal microbiology (Caution: not perioperative) |
|  | Meropenem/ Vancomycin orally | Suspicion of Clostridium infection |
|  | Administration of antihistamines | Suspicion of allergic reaction/anaphylaxis |
|  | Drug reaction (ADR, ADE) | Rash, itching, hypotension, allergic reaction |
|  | Administration of vitamin K, administration of protamine, administration of antagonizing medications, overdose (e.g., naloxone administration) | Coagulation abnormalities, liver failure, bleeding |
|  | Drug level outside the therapeutic range | Dosing error, administration error, formulation error, insufficient drug onset |
|  | Administration of antiemetic | In case of nausea and/or vomiting, overly rapid advancement of feeding, medication-related, allergic reaction |
|  | Administration of a fluid bolus | Crystalloid/colloid administration for volume depletion, hypotension, coagulation abnormalities, electrolyte imbalance for correction |
|  | Administration of laxatives/ prokinetic agents | Constipation due to opioid/analgosedation overdose, volume depletion |
|  | Administration of coagulation products (procoagulants) | Coagulation abnormalities, volume depletion, resuscitation, FFP, platelet concentrates, PCC, tranexamic acid |
|  | Administration of insulin and glucagon | In case of blood glucose imbalance, hyperkalaemia |
|  | Administration of anticonvulsants | In case of newly onset seizures during the hospital stay |
|  | Additional (as-needed) administration of calming, sedative medication | Inadequate treatment regimen for delirium and withdrawal, agitation, anxiety, confusion (additional sedation) |
|  | Unplanned, abrupt discontinuation of medication | Indication of inadequate care, medication reaction or interaction, indication of a change in the patient’s condition/modified treatment regimen |
|  | Increased pain level | Pain exacerbations requiring rescue medication |
| **Procedural and Care** | Transfer to a higher level of care | IMC/ICU transfer, unplanned postoperative ICU admission, return transfer to ICU within 48 h |
|  | Readmission/ return to hospital (within 30 days after discharge) | inadequate treatment or diagnostic workup |
|  | Inadequate care (disregard of clinical/diagnostic/laboratory findings, insufficient scoring, inadequate response to signs), medical failure | Disregard of clinical/diagnostic/laboratory findings, insufficient scoring, inadequate response to signs |
|  | Change in procedure or treatment plan | Miscommunication, staff turnover, response to clinical/therapeutic course |
|  | Delayed/postponed procedure, delayed discharge/transfer | Poor management, organizational issues |
|  | Treatment-related error | Wrong patient, wrong assignment, wrong side, wrong surgery |
|  | Unplanned reoperation | in case of a complication: unsatisfactory surgical outcome, effusion, retention |
| **ICU Module** | Initiation of catecholamines (> 24 h postoperatively) | Hypotension, vasoconstriction, impaired organ perfusion, sepsis |
|  | Administration of sodium bicarbonate | Treatment of metabolic acidosis, intoxication |
|  | Intubation, reintubation, tracheotomy, cricothyrotomy (within 24 h after extubation), accidental extubation | Failed extubation due to premature, inadequate weaning, laryngeal injury, disregard of clinical signs, deterioration, infection |
